# Experience with two antimicrobial prescribing tools in ambulatory care settings

**DOI:** 10.1017/ash.2022.278

**Published:** 2022-08-11

**Authors:** Zahra Kassamali Escobar, Scott Thomasson, Todd Bouchard, Staci Kvak, Kyung Min Lee, Jose Mari Lansang, John B. Lynch, Larissa May, Marisa D’Angeli, Chloe Bryson-Cahn

**Affiliations:** 1 University of Washington Medicine, Valley Medical Center Renton, Washington; 2 University of Washington School of Pharmacy, Seattle, Washington; 3 University of Washington Medical Center, Seattle, Washington; 4 Division of Allergy and Infectious Diseases, University of Washington School of Medicine, Seattle, Washington; 5 Department of Emergency Medicine, University of California–Davis Health, Sacramento, California; 6 Healthcare Associated Infections Program, Washington State Department of Health, Shoreline, Washington

## Abstract

We compared experiences with The Multifaceted Intervention to Improve Prescribing for Acute Respiratory Infection for Adult and Children in Emergency Department and Urgent Care Settings versus Choosing Wisely to evaluate inappropriate antimicrobial prescribing in ambulatory care. Both identified the same clinics, diagnoses, and antibiotics for high-yield antibiotic stewardship interventions.

Inappropriate antibiotic prescribing for viral respiratory tract infections (RTIs) is an important target for antimicrobial stewardship^1^; up to 45% of outpatients with an RTI are prescribed antibiotics.^2–4^ Multiple tools have been developed to evaluate antibiotic prescribing in ambulatory settings. The Multifaceted Intervention to Improve Prescribing for Acute Respiratory Infection for Adult and Children in Emergency Department and Urgent Care Settings (MITIGATE) and Choosing Wisely are 2 such tools promoted by the Centers for Disease Control and Prevention (CDC) and the American Board of Internal Medicine (ABIM), respectively. MITIGATE and Choosing Wisely use *International Classification of Disease, Tenth Revision* (ICD-10) diagnosis codes and associated antibiotic prescriptions to identify rates of inappropriate prescribing for viral RTI.^5,6^ The principal difference between Choosing Wisely and MITIGATE is that MITIGATE includes diagnoses for which antibiotics are never appropriate whereas Choosing Wisely includes diagnoses for which antibiotics are never appropriate and diagnoses for which antibiotics are sometimes appropriate. Therefore, the target antibiotic prescribing rate is zero for MITIGATE but an undefined, nonzero value for Choosing Wisely. In 2017, prior to publication of the MITIGATE tool kit, we implemented Choosing Wisely in our urgent-care clinics. Although sharing data helped identify prescribing outliers, it was difficult to gain consensus around a target antibiotic prescribing rate for RTI. In 2019, we pivoted toward MITIGATE and scaled to more clinics and clinicians. We maintained the Choosing Wisely database to confirm that MITIGATE was capturing most RTIs and to identify any diagnostic shift, that is, whether clinicians would deliberately select antibiotic appropriate diagnoses to maintain a low rate of unnecessary antibiotic prescribing per MITIGATE criteria. We compared experience with each of these frameworks and the data they yielded.

## Methods

This observational investigation of antibiotic prescribing for viral RTI in ambulatory care settings was conducted at the UW Medicine Valley Medical Care in Renton, Washington. Data were collected between March 1, 2018, to April 30, 2019, prior to implementing stewardship interventions of the MITIGATE tool kit.^6^ The Choosing Wisely data set captured 5 urgent care clinics, 11 primary care clinics, and 25 specialty care clinics. The MITIGATE data set included the same clinics plus 1 additional primary care clinic and the emergency department (ED) of Valley Medical Center, a 341-bed, acute-care hospital and level-3 trauma center. Between the time we implemented Choosing Wisely and MITIGATE, an additional primary care clinic opened and ED engagement was achieved. As a result, we included both in MITIGATE but not in Choosing Wisely. Viral RTI were defined according to ICD-10 codes identified by the respective tool kits.^4–6^ MITIGATE identified 24 diagnoses for viral RTIs, which translated to 157 ICD-10 codes, and Choosing Wisely included 45 ICD-10 diagnosis codes. In the MITIGATE data set, patients with competing diagnoses or comorbidities that might indicate appropriate antibiotic prescribing (eg, a concomitant urinary tract infection) at the time of the RTI visit were excluded. Only antibiotics prescribed on the visit date were included. In the Choosing Wisely data set, patients with competing diagnoses 30 days prior or 7 days after the index visit or comorbid diagnoses within 12 months of the index visit were excluded. Prescribed antibiotics were captured between day 0 and up to 3 days after the index visit. The primary outcome for both data sets was rate of antibiotic prescribing. The Institutional Review Board of the University of Washington approved the study and waived written informed consent.

## Results

**Fig. 1.**
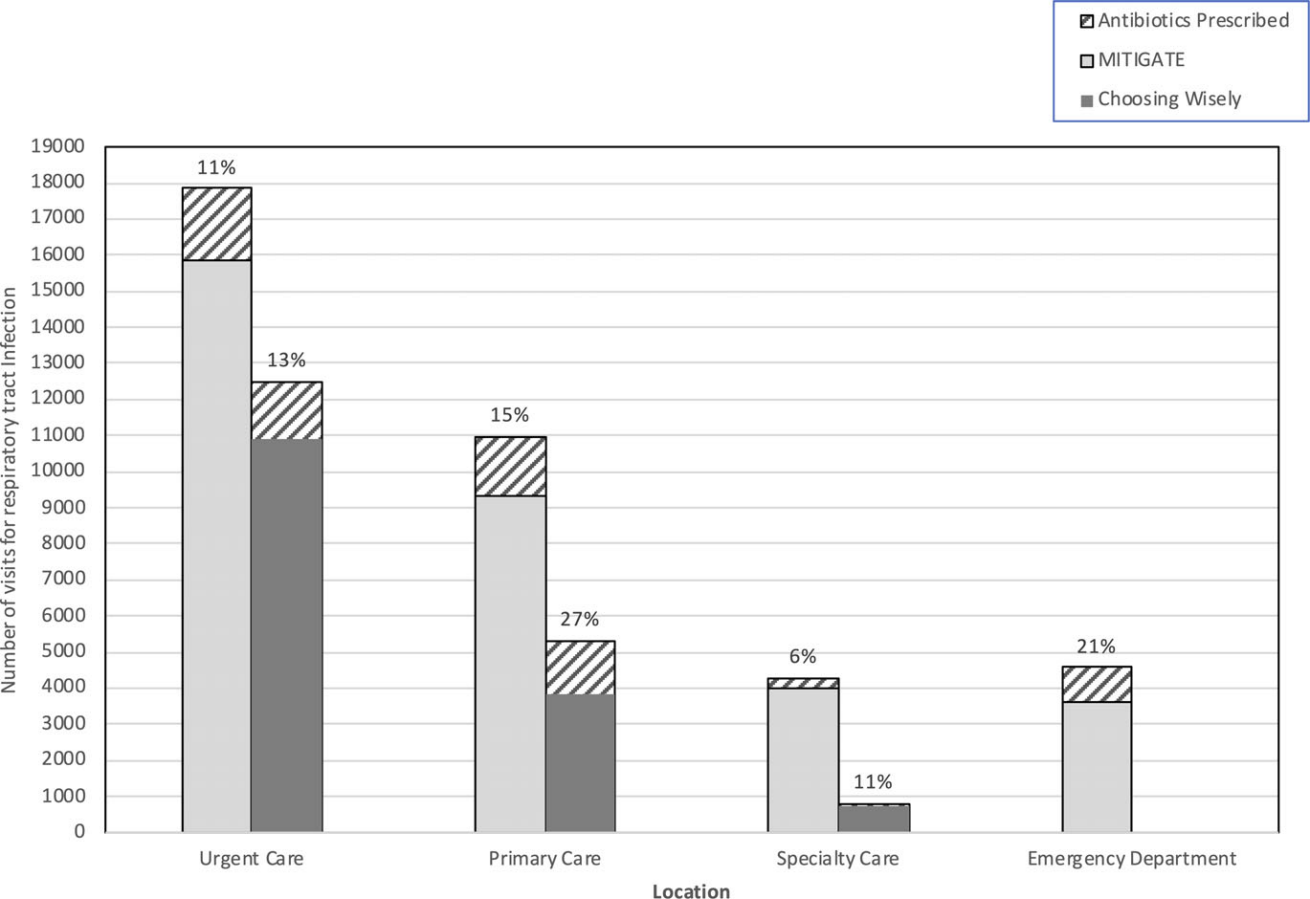
Respiratory tract infection visits and proportion receiving antibiotics in the MITIGATE and Choosing Wisely data sets, March 1, 2018, through April 30, 2019. Population differences between stewardship frameworks include addition of a primary care clinic and the ED in MITIGATE but not Choosing Wisely.

During a 13-month period, 37,661 patient visits met MITIGATE inclusion criteria and 10% received an antibiotic. In the same period, 18,599 patient visits met Choosing Wisely inclusion criteria and 17% received an antibiotic. Urgent care was the site most commonly visited for RTI and accounted for 17,844 visits (47%) in the MITIGATE data set and 12,509 visits (67%) in the Choosing Wisely data set. Antibiotic prescribing was highest in the ED (21%) and lowest in specialty care (6% in MITIGATE and 13% in Choosing Wisely) (Fig. 1). The most frequently coded diagnosis in both frameworks was acute respiratory infection, with unspecified (J06) accounting for 31% and 55% of visits each in MITIGATE and Choosing Wisely. In MITIGATE, the indication with antibiotics most frequently prescribed was lower RTI (Table 1). In Choosing Wisely, the indication most frequently prescribed antibiotics was sinusitis, and notably, sinusitis was excluded from MITIGATE. Azithromycin was the antibiotic most commonly prescribed: 2,062 (43%) of 4,823 prescriptions in the MITIGATE data and 1,118 (33%) of 3,380 prescriptions in the Choosing Wisely data. Amoxicillin-clavulanate and amoxicillin accounted for 11% and 21% of antibiotic prescriptions, respectively, in MITIGATE data and 25% and 21% of antibiotic prescriptions, respectively, in Choosing Wisely data. Fluoroquinolone prescriptions were infrequent: 140 (3%) in MITIGATE data and 88 (3%) in Choosing Wisely data.

**Table 1.** Antibiotic Prescribing by Diagnosis. The number of visits for respiratory tract infections and percentage with antibiotics prescribed are shown according to the MITIGATE and Choosing Wisely Frameworks, ranked by diagnoses with the highest rate of antibiotic prescribing. Population differences between stewardship frameworks include addition of a primary care clinic and the ED in MITIGATE but not Choosing Wisely.

MITIGATE			Choosing Wisely		
ICD-10 Code & Description	Antibiotic Prescriptions/Visits for Specified Diagnosis	%	ICD-10 Code & Description	Antibiotic Prescriptions/Visits for Specified Diagnosis	%
J22, Lower RTI, unspecified	139/188	74	J01 Sinusitis	1,823/2,486	73
H65 Nonsuppurative otitis media	1,034/1,702	61	J20 Acute bronchitis	380/2,200	17
J40 Bronchitis, NOS	1,307/3,185	41	R05 Cough	919/6,556	14
J12 Viral pneumonia	4/11	36	J06 Acute URI of multiple and unspecified sites	478/15,424	**3**
J20 Acute bronchitis	638/3,150	20	J10 Influenza due to other influenza virus	16/888	**2**
J45 Asthma	926/10,911	8	J11 Influenza due to unidentified influenza virus	18/1,302	1
J31 Chronic rhinitis, nasopharyngitis, and pharyngitis	74/922	8			
J06 Acute URI^c^ of multiple and unspecified sites	758/11,004	7			
J04 Laryngitis, laryngotracheitis	37/565	7			
J21, Bronchiolitis	32/508	6			

Note. ICD-10, *International Classification of Disease, Tenth Revision*; RTI, respiratory tract infection; URI, upper respiratory infection; NOS, not otherwise specified.

## Discussion

Rates of antibiotic prescribing for viral RTI varied between the tool-kit definitions used, related to the diagnosis codes included and excluded and differences in the groups sampled. Nevertheless, both frameworks flagged urgent care and primary care as locations where most patients with RTI are seen and where antibiotics for these indications are commonly prescribed. Both identified azithromycin as the most prescribed antibiotic for RTI. In the same timeframe, MITIGATE produced a sample size much larger than the Choosing Wisely data. This was unexplained by the addition of a primary care clinic and the ED. Even in urgent care where the sampled sites were the same, 5,335 (43%) more visits were captured by MITIGATE than by Choosing Wisely.

We previously described the significant amount of support required to build these data sets^7^; one was not easier to build than the other. Although the technical specifications revealed the same trends, the end-user experiences differed. The lack of a clear benchmark with Choosing Wisely made a target prescribing goal difficult to determine and therefore enforce. This nuance may make Choosing Wisely more appropriate for institutions that treat patients who would be excluded from the MITIGATE data set, such as patients with HIV, active malignancy, or transplant history. MITIGATE is prescriptive regarding stewardship interventions to accompany antimicrobial use data and includes suggested e-mail text to communicate the data to individual prescribers.^4^ Choosing Wisely simply provides the data, citations for expert guidelines, and leaves intervention up to the end user.^5^ Another benefit of MITIGATE is its transparency and availability of technical specifications on an Open Access platform.^4^

A primary concern for using the MITIGATE tool kit was the exclusion of diagnoses that are sometimes antibiotic appropriate. Having both data sets allowed evaluation of any diagnostic shift, that is, prescribers selecting respiratory infection diagnoses that sometimes warrant antibiotics not captured by the MITIGATE tool kit. In the urgent care setting, this seems unlikely; 11% versus 13% antibiotic prescribing is not substantially different. In the primary care setting, however, antibiotic prescribing was almost twice that in the Choosing Wisely data set (27%) compared to MITIGATE (15%), which merits further exploration.

This study had several limitations. This study was conducted at a single site and did not include evaluation of other stewardship tools. There were differences in clinics selected. Because we did not apply the Choosing Wisely framework to the ED, we could not determine whether MITIGATE fully captured the scope of antibiotic prescribing. Finally, the MITIGATE analysis only captured antibiotics prescribed on the date of visit, therefore nonvisit antibiotic prescriptions were not included.

In our experience at a community healthcare system without a substantial immunocompromised patient population, MITIGATE was preferable to implement antimicrobial stewardship for RTI. Benefits of this tool kit include public availability of the technical specifications and an accompanying manual of how to implement stewardship interventions. Concerns that MITIGATE might not capture all RTIs or might yield an incorrect snapshot of antibiotic prescribing due to diagnostic shift was unfounded in the urgent care clinics. However, primary care clinics and the ED are areas to explore further.
